# The localization of the alkaloids in *Coptis chinensis* rhizome by time-of-flight secondary ion mass spectrometry

**DOI:** 10.3389/fpls.2022.1092643

**Published:** 2022-12-23

**Authors:** Fan He, Yu-Feng Huang, Wei Dai, Xian-You Qu, Jing-Guang Lu, Chi-Chou Lao, Wen-Hui Luo, Dong-Mei Sun, Mei Wei, Sheng-Yuan Xiao, Ying Xie, Liang Liu, Hua Zhou

**Affiliations:** ^1^ Guangdong Provincial Academy of Chinese Medical Sciences, Guangdong Provincial Hospital of Chinese Medicine, Guangzhou, Guangdong, China; ^2^ Institute of Chinese Medicinal Materials, Mianyang Academy of Agricultural Sciences, Mianyang, Sichuan, China; ^3^ Chongqing Key Laboratory of Traditional Chinese Resources, Chongqing Academy of Chinese Materia Medica, Chongqing, China; ^4^ Faculty of Chinese Medicine and State Key Laboratory of Quality Research in Chinese Medicine, Macau University of Science and Technology, Taipa, Macao SAR, China; ^5^ Guangdong Provincial Key Laboratory of Traditional Chinese Medicine Formula Granule, Guangdong Yifang Pharmaceutical Co., Ltd., Foshan, Guangdong, China; ^6^ College of Chinese Medicinal Materials, Jilin Agricultural University, Changchun, Jilin, China

**Keywords:** *Coptis* rhizome, TOF-SIMS, UPLC-QQQ-MS/MS, alkaloids, localization

## Abstract

**Background:**

Understanding the spatial distribution of active compounds can effectively evaluate the quality of decoction pieces of traditional Chinese medicine (TCM). Traditional methods are economical and practical but lack chemical information on the original distribution. Time-of-flight secondary ion mass spectrometry (TOF-SIMS), with the advantage of non-destructive detection of samples, can directly analyze the distribution of chemical compounds on the surface of various samples.

**Methods:**

In this study, TOF-SIMS image analysis technology was used to detect TCM for the first time. Taking Coptis rhizome (CR) as an example, a commonly used TCM, the distribution of the compounds in the cross-section of CR was studied. Meanwhile, ultra-high-performance liquid chromatography coupled with triple quadrupole mass spectrometry (UPLCQQQ-MS/MS) was used to verify the results of TOF-SIMS.

**Results:**

The distribution of nine active compounds: berberine, epiberberine, coptisine, palmatine, columbamine, jatrorrhizine, tetrahydricheilanthifolinium, and oxyberberine, was well imaged in the cross-section of CR by TOF-SIMS. The content of berberine and epiberberine was the highest; Palmatine distribution in the pith was more than that in other parts; Oxyberberine was mainly concentrated in the cork and xylem rays. Normalization analysis showed contents of these compounds increased along with the growth years. The result was consistent with UPLC-QQQ-MS/MS.

**Conclusion:**

The TOF-SIMS method can display the spatial distribution status of the active compounds of herbs, providing a basis for selecting the medicine site with non-destructive and fast detection.

## 1 Introduction

For thousands of years, traditional Chinese medicine (TCM) has shown unique advantages in preventing and curing diseases and has made outstanding contributions to the health of Chinese people. The quality evaluation of medicinal materials is critical to ensure the ideal effect of medicinal materials. Character identification is a traditional way to evaluate the quality of TCMs (Wei et al., 2021). It mainly judges the authenticity of TCMs through the morphological characteristics of the medicinal materials, including the features of shape, color, smell, and texture. The character identification method is simple and fast but subjective and based on experience. These shortcomings severely limit their application. Combining the characteristics and advantages of TCM with modern science and technology and developing modern methods suitable for TCM research have become urgent and critical tasks. The current quality evaluation of TCM draws on the quality control scheme of chemical medicines. It applies modern instrumental analysis methods, such as chromatography, mass spectrometry (MS), or combining the two techniques (Zhao et al., 2012). It usually employs active or primary chemical ingredients as markers for qualitative and quantitative analysis. However, these methods do not accurately locate the chemical components and lack the spatial distribution of ingredients in the tissue cells.

Mass spectrometry imaging (MSI) obtains metabolite signals from the surface of biological or tissue samples through mass spectrometry detection (Anderson et al., 2010; Buchberger et al., 2018). In addition, it can simultaneously obtain an overall visual-spatial distribution map of chemical components in the organism through computer imaging. The application of MSI to the rapid positioning of specific chemical components of medicinal plants is expected to achieve a breakthrough in evaluating medicinal properties (Bjarnholt et al., 2014).

According to different ionization methods, MSI technology can be divided into matrix-assisted laser destruction ionization mass spectrum imaging (MALDI-MSI), destruction electrospray ionization mass spectrum imaging (DESI-MSI), secondary ion mass spectrum imaging (SIMS), and so on. From the perspective of analysis objects, MALDI-MSI applies to the analysis of proteins, polypeptides, phospholipids, and other substances with large molecular weights (Caprioli et al., 1997; Stoeckli et al., 2001; Jackson et al., 2005). DESI-MSI applies to the analysis of small molecular metabolites (Wu et al., 2013; Banerjee et al., 2017; Sun et al., 2019). SIMS applies not only to element analysis but also the analysis of small organic molecules with a molecular weight below 1000 Da (Todd et al., 1997; Passarelli et al., 2017).

SIMS has a label-free, highly sensitive, multi-component analysis detection function (Lockyer, 2014). Also, it has sub-micron or even nano-level high spatial resolution imaging capabilities. The principle of time-of-flight secondary ion mass spectrometry (TOF-SIMS) detection is based on the phenomenon that a primary ion beam with an energy of several thousand electron volts bombards the surface of a solid sample (Barreto et al., 2014). After physical interaction, secondary ions are generated on the sample’s surface.

The early SIMS was mainly used for material analysis in the industry. With the advent of the biological century, SIMS has been used in many research fields of life sciences. TOF-SIMS was mainly used to detect animal tissues such as the brain, liver, small intestine, and skeletal muscle. It compared the characteristics of endogenous substances such as lipids and cholesterol in health and diseases and intuitively reflected the characteristics of diseases. TOF-SIMS has also been successfully applied to study the spatial distribution of metal ions and active ingredients in plant tissues (Metzner et al., 2008; Zhou et al., 2011; Jung et al., 2012; Kuroda et al., 2013; Fu et al., 2018). The application characteristics of TOF-SIMS can make up for the inability to accurately locate the chemical components in TCM and the lack of the spatial distribution of chemical components in tissue cells. However, there is no research report on TOF-SIMS in TCM so far. Therefore, we took the CR as an example for the first time to introduce TOF-SIMS technology in the detection of TCM in this study.

Coptis Rhizome (CR) is the dried rhizome of *Coptis chinensis* Franch., *Coptis deltoidea* C.Y.Cheng et Hsiao., and *Coptis teeta* Wall. in the Chinese pharmacopoeia (Pharmacopoeia Commission of PRC, 2020). Modern studies have shown that the pharmacological effects of CR are mainly related to the alkaloids in its rhizome. CR and its chemical components in clinics are often used to treat dysentery, diabetes, insomnia, etc. (Wang et al., 2019). Traditionally, CR with the thick rhizome, the orange-red phloem, and the right or orange-yellow xylem is considered high-quality (Yang et al., 2017). To clearly understand the significance of these features, some researchers have used freehand or microdissection to separate the tissues of CR and used spectroscopy, HPLC, and LC-MS to detect the alkaloid content in each cell or different tissues (Zhao et al., 2011; Yi et al., 2015; Yang et al., 2017; Wang et al., 2020). Although these methods could correlate external characteristics with internal quality, they need to cut, enrich and extract samples. Many interference factors in the operation cannot directly reflect the relationship between traditional traits and modern active ingredients.

In this study, TOF-SIMS technology was first introduced to detect Chinese herbal medicine. Taking a fresh medicinal product with different growth years as an example, TOF-SIMS was used to locate the spatial distribution of chemical components in CR. For the first time, the *in-situ* positioning method was used to analyze the distribution of the nine alkaloids in the tissue of CR, correlated the appearance characteristics with the intrinsic medicinal value, and provided a new detection method for the quality control of Chinese medicinal materials.

## 2 Materials and methods

### 2.1 Herbal materials and chemicals

**Figure 1:**
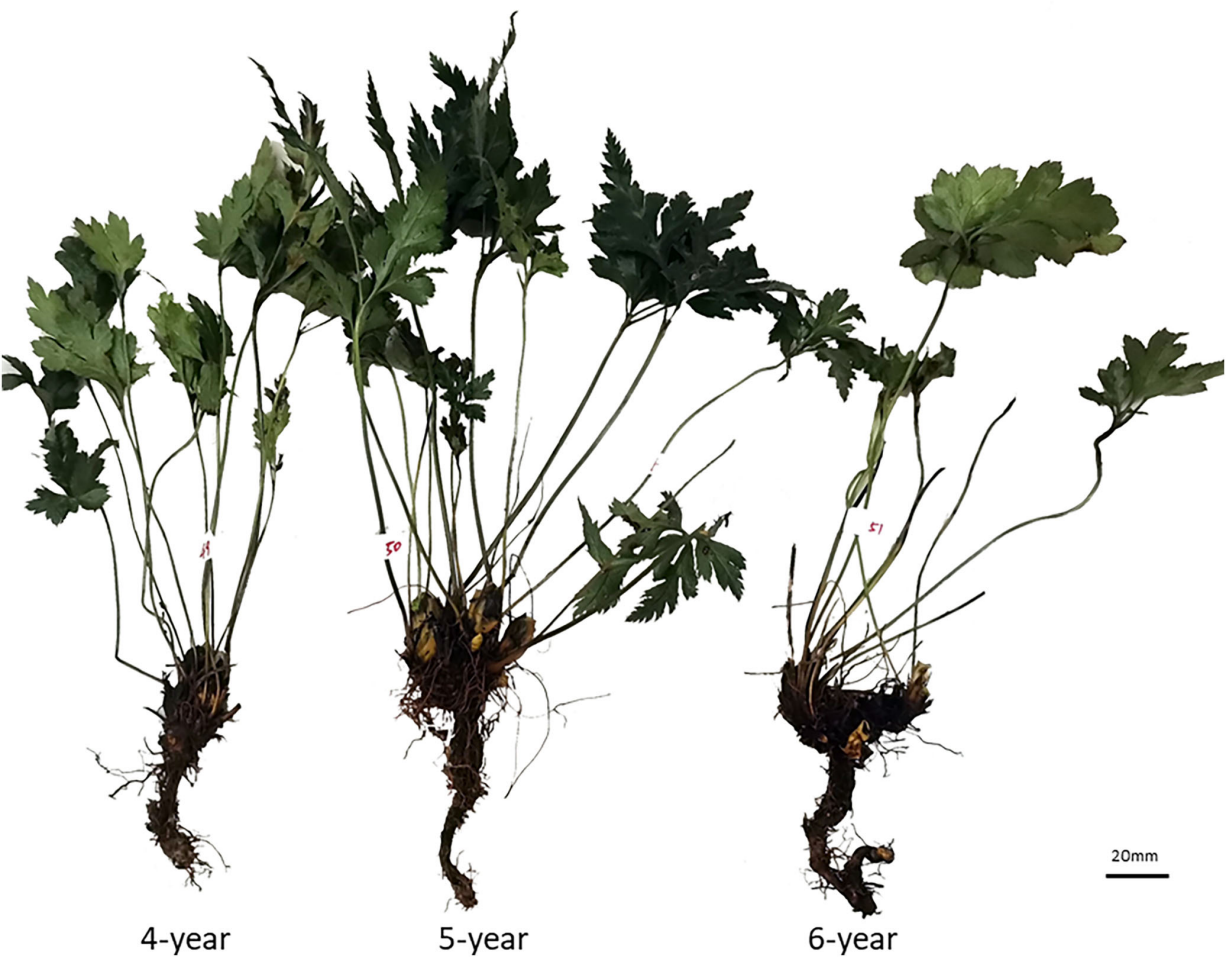
The plant morphology of *Coptis chinensis* rhizome.

**Figure 2:**
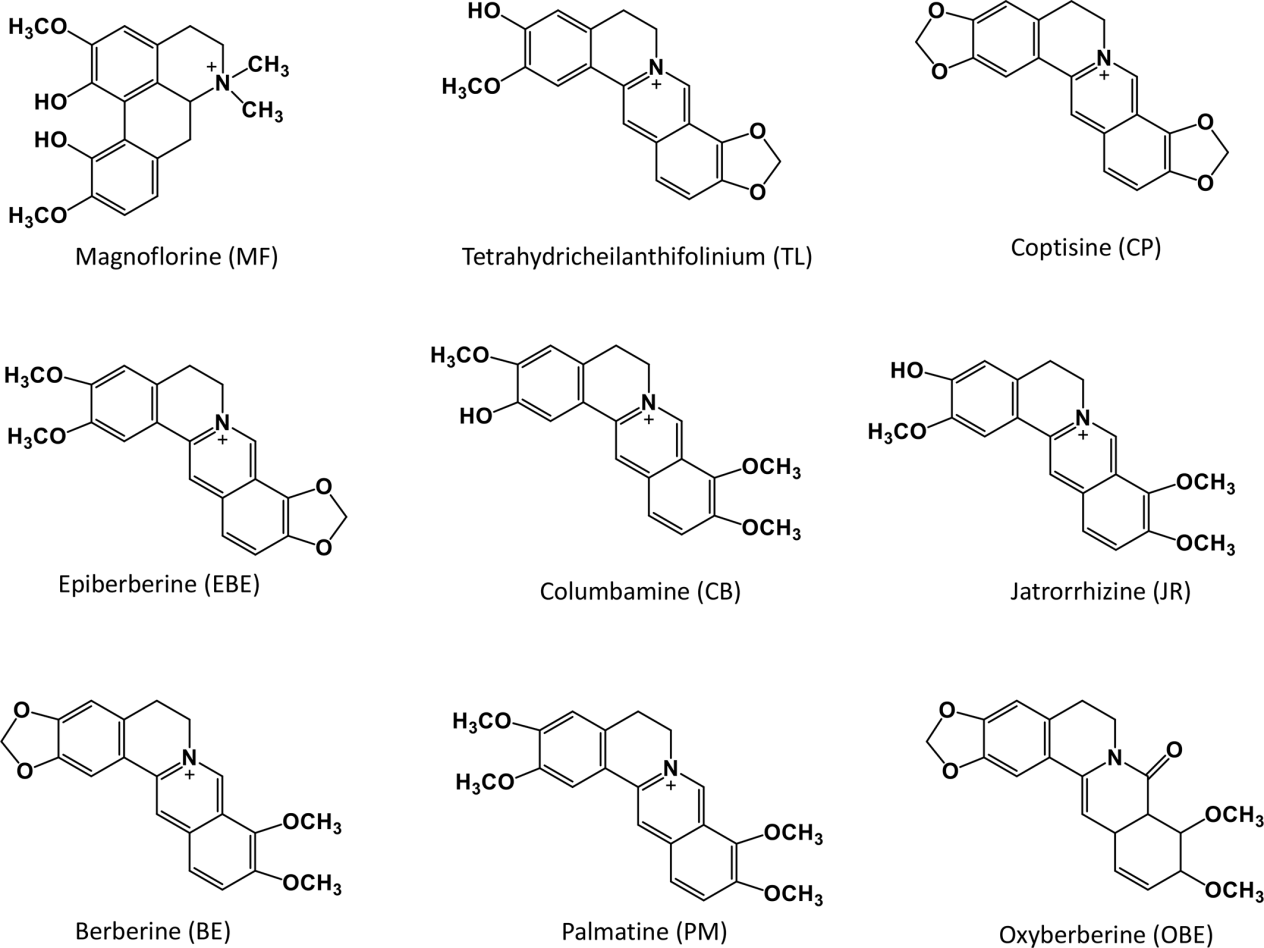
Chemical structure of nine compounds in *Coptis chinensis* rhizome.

The whole fresh CR samples were collected from the same area of Mianyang City, Sichuan Province. Prof. Hua Zhou authenticated all the plant materials. The plant morphology is shown in **Figure 1**. All specimens are stored in the State Key Laboratory of Quality Research in Chinese medicine (Macau University of Science and Technology). All the reference standards (purity ≥98%) were purchased from Shanghai Yuanye Bio-Technology Co., Ltd. (Shanghai, China). The chemical structures of these compounds are shown in **Figure 2**. The UPLC grade acetonitrile, methanol, and formic acid was obtained from Fisher Scientific (Pittsburgh, PA, USA). The other chemical reagents were of analytical grade. The water was obtained from a Milli-Q water purification system (Millipore, Bedford, MA, USA).

### 2.2 Tissue preparation

The washed fresh CR was cut into 0.5 cm sections and moved into a suitable box with optimal cutting temperature compound (OCT, Leica Microsystems, Wetzlar, German) embedding agent. After soaking for 20 minutes, these samples were moved to a refrigerator at -20°C and froze for 30 minutes. The sample was fixed on the cryostat stage (Leica CM 1950, German). After adjusting the position, it was cut into slices with a thickness of 10μm at -22°C. The slices were directly attached to the glass and the silicon at room temperature and dried under a vacuum.

### 2.3 Optical microscopy

The glass patch was placed on the stage to keep it flat and fixed. The light source of the microscope was turned on, and the 4 × objective lenses were selected first. After adjusting and selecting an appropriate field of view, the image was recorded by the digital camera Olympus on the IX73 microscope. The microscope’s light source was turned off, and then the fluorescent light source was turned on. The light source selection button was dialed to DAPI (blue light), FITC (green light), and TRITC (red light) in turn. The related parameters of the microscope were adjusted, and the image was recorded.

### 2.4 TOF-SIMS analysis

TOF-SIMS analyses were performed on a TOF-SIMS IV (ION-TOF GmbH, Münster, Germany) instrument equipped with a Bi ion gun (LMIG) and a reflection electron analyzer with a multi-channel detector. The mass spectrum and ion density map were collected using the 30 keV Bi^3+^ primary ion (〜0.4 pA) at 45° incidence and low-energy electron flooding for charge compensation. Large-area imaging was performed using the traditional high-current beam-forming focusing mode, where the current measured at 10 kHz was 0.40 pA. The imaged area was 9500 μm × 7000 μm divided by 950 pixels × 128 pixels, resulting in a spatial resolution of about one micrometer. The ion dose density applied was 1.25 × 10^9^ ions/cm^2^. The Bi^3+^LMIG was operated at a cycle time of 100 μs (mass range: 0~960 u). A low-energy pulsed electron gun (21 eV) neutralized the charge on the ample insulating surface. Data processing was performed using SurfaceLab 6.7 (ION-TOF GmbH, Münster, Germany). Positive spectra were mass-calibrated using CH_3_^+^, C_2_H_3_^+^, C_2_H_5_^+^, C_3_H_5_^+^, C_4_H_7_^+^, C_5_H_9_^+,^ C_10_H_19_^+^, C_15_H_29_^+^ and C_20_H_39_^+^. The attainable mass resolution was ~4000 at m/z 29 (C_2_H_5_^+^). The TOF-SIMS analysis was performed three times for each specimen to confirm the repeatability and biological reproducibility of the study.

Regardless of the specific ion or the total ion image, the ion image was not normalized. Ion images were all recorded with the same primary ion dose so that they could be directly compared. The only influencing factor was that the matrix effect may always exist, causing the ion emission of a particular ion to increase or decrease due to its microenvironment. However, this did not affect the relative comparison of the emission of specific ions between the histologically available areas.

In the secondary ion image, the compound name or the m/z value of the corresponding icon, the maximum count (MC) in the pixel, and the total count (TC) are displayed below each image (except for color overlay). Thus, the color scale corresponds to the interval [0, MC].

### 2.5 Standards solutions

The standards were accurately weighed and dissolved in methanol individually as a stock solution. Nine kinds of standard stock solutions were used to prepare a mixed standard solution with a concentration of 6.34 μg/mL for Magnoflorine (MF), 5.38 μg/mL for tetrahydricheilanthifolinium (TL), 36.5 μg/mL for coptisine (CP), 34.7 μg/mL for epiberberine (EBE), 5.82 μg/mL for columbamine (CB), 12.35 μg/mL for jatrorrhizine (JR), 32.3 μg/mL for berberine (BE), 27.3 μg/mL for palmatine (PM), and 2.86 μg/mL for oxyberberine (OBE). Then, a series of working solutions were obtained by serially diluting the two-speed stock solution with methanol to obtain the desired concentration.

### 2.6 Sample extraction

The sample was extracted according to the previously published method. [13] Briefly, weighed accurately 0.2 g of the powder of CR (through 24 mesh) to a stoppered conical flask and accurately added 50 mL of a mixture of methanol and hydrochloric acid (100:1). Weighed and ultrasonicated (power 250 W, frequency 40 kHz) for 30 min. Then, replenished the solvent loss with methanol and filtered. Measured precisely 2 mL of the successive filtrate to a 10dmL volumetric flask, diluted it with methanol to volume, and used the successive filtrate as the test solution.

### 2.7 Liquid chromatography-mass spectrum analysis

Chromatographic analysis was performed on an Agilent1290 series UPLC system (Agilent Technologies, Santa Clara, CA, USA) equipped with a binary pump, an online degasser, an autoplay sampler, and a monitoring column thermostat. Chromatographic separation was carried out at 30°C on a Waters Acquity UPLC C_18_ column (2.1×100 mm, 1.7 μm) (Waters, Milford, MA, USA). A mobile phase was consisting of 0.1% acetic acid (A) and acetonitrile (B) in a gradient elution manner as following: 10-14% B at 0-3 min, 14-16% B at 3-9 min, 16-25% B at 9-13 min, 25-80% B at 13-14 min, 80% B at 14-16 min. The re-equilibration time of gradient elution was 2 min between each run. The sample injection volume was 2 μL, and the flow rate was 0.35 mL/min. MS detection was performed on an Agilent 6460 Triple Quadrupole Mass Spectrometer (Agilent Technologies, Santa Clara, CA, USA) equipped with an electrospray ionization (ESI) source. The mass spectrum was chosen in positive mode, and the MS spectra were acquired in MRM mode. The drying gas (N_2_) flow rate was 11.0 L/min; the drying gas temperature was 300°C; the nebulizer was 15 psig, and the capillary voltage was 4000 V. The precursor ion, production, fragment, and collision energy (CE) were adjusted to obtain the highest abundance of each analyte (**Table 1**).

**Table 1:** The multiple reaction monitoring (MRM) parameters and collision energy of nine target markers in the UPLC-QQQ-MS/MS analysis.

No.	Compound name	precursor ion	product ion	Fragment(V)	collision energy(V)
1	Magnoflorine(MF)	343.2	166.1	130	76
2	Tetrahydricheilanthifolinium(TL)	323.1	308.1	160	28
3	Coptisine (CP)	321.1	293.2	160	28
4	Epiberberine EBE)	337.1	321.1	160	32
5	Columbamine (CB)	339.2	323.2	170	28
6	Jatrorrhizine (JR)	339.2	323.2	140	28
7	Berberine (BE)	337.1	321.1	160	28
8	Palmatine (PM)	353.2	337.2	170	28
9	Oxyberberine (OBE)	352.1	322.1	160	32

### 2.8 Method validation

The calibration curves for analytes were constructed by the peak area of the standard (*y*-axis) against the concentration of the standard (μg/mL; *x*-axis). The limit of detection (LOD) and limit of quantification (LOQ) for each analyte were measured by diluting the lowest concentration standard solution to a signal-to-noise ratio (S/N) ratio of 3 and 10, respectively.

The method was validated according to the Chinese Pharmacopoeia Edition 2020 [13]. The precision was detected in one mix standard solution six times in a single day. The stability was analyzed in a sample solution after placing 0, 2, 4, 6, 8, and 12 h at room temperature. The repeatability was determined by six independent samples prepared in parallel. The recovery was validated by adding a certain amount (100% of the known amount) of each standard solution to samples six times. All samples were extracted and analyzed as described above. The recovery of 10 analytes was calculated as follows: Recovery (%) = 100 × (the amount found − original amount)/amount spiked.

## 3 Results

### 3.1 Visible characteristics of each sample

**Figure 3:**
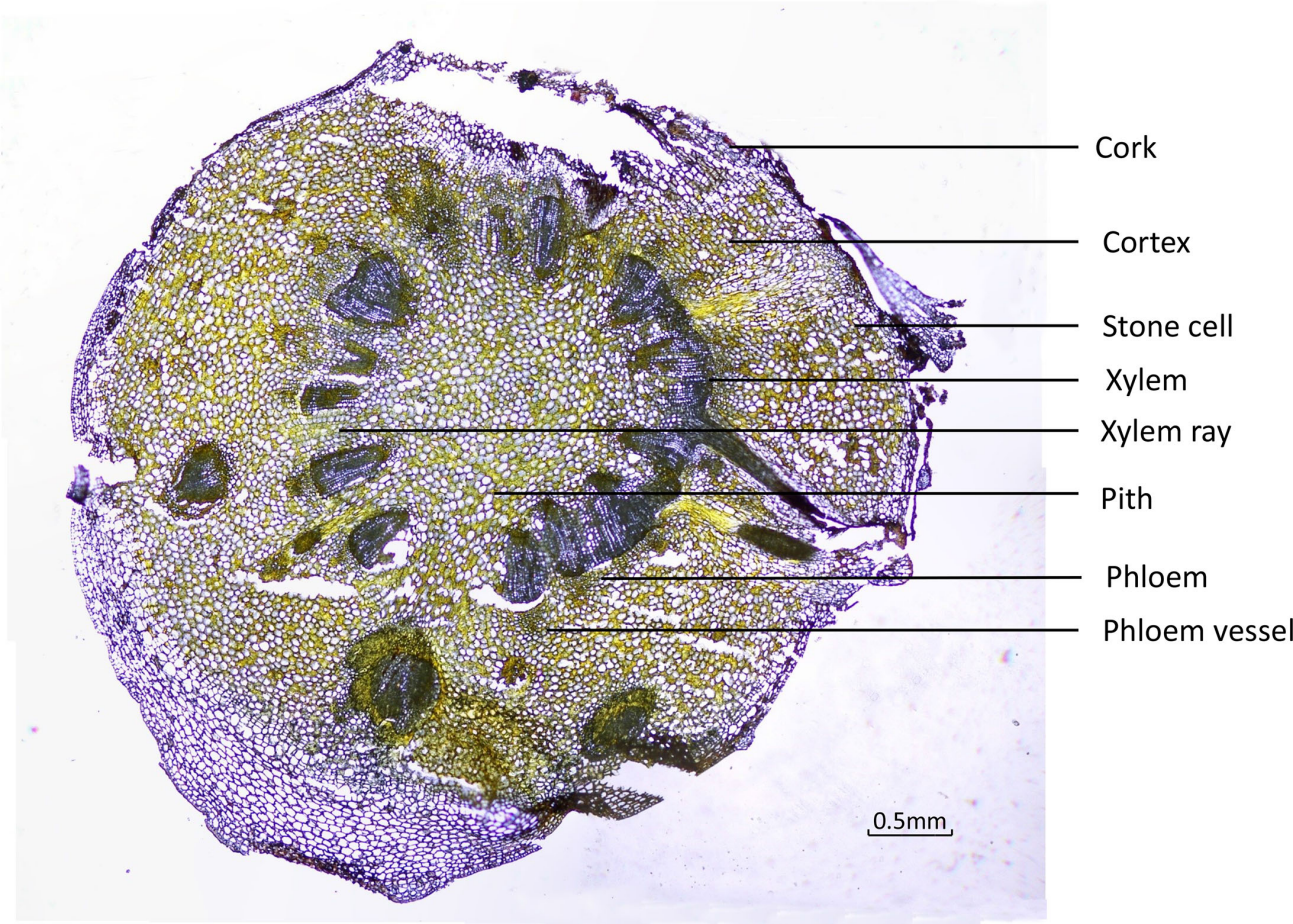
Transverse sections of *Coptis chinensis* rhizome observed under a light microscope (40×).

To ensure that the different tissues were identified from the fresh roots, cross-sections were examined under the microscope using normal light and fluorescence mode. Under ordinary microscopy observation, as in **Figure 3**, the cork layer was composed of several rows of cells, and there was a cork outside the cork layer, which often fell off. The cortex was wide, and the stone cells were scattered individually or in groups. The pericyclic were bundled or accompanied by a few stone cells, which were all yellow. The vascular bundles were externally tough and ring-shaped. The xylem was yellow, all woody, and the wood fiber was more developed. The pith was parenchyma cells without stone cells.

**Figure 4:**
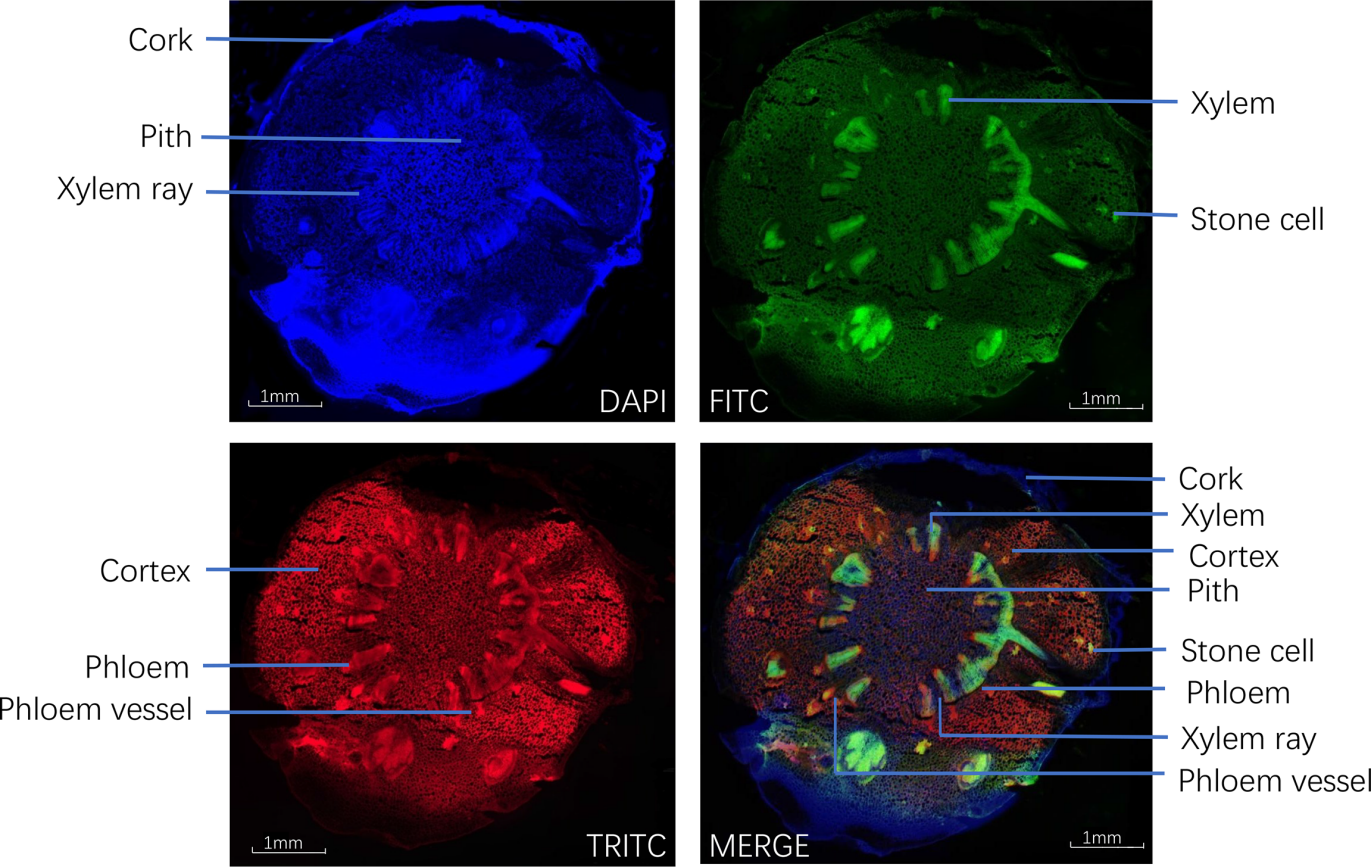
Transverse sections of *Coptis chinensis* rhizome observed under a fluorescence microscope (40×).

Under fluorescence microscopy, the cross-section of CR showed different auto-fluorescence colors under different filters in fluorescence mode. The cork, xylem ray, and pith of CR could be observed in DAPI mode; the stone cells and xylem in CR could be clearly observed in FITC mode; the cortex, phloem, and phloem vessel could be seen more clearly in TRITC mode (**Figure 4**).

### 3.2 Chemistry mapping of TOF-SIMS

**Figure 5:**
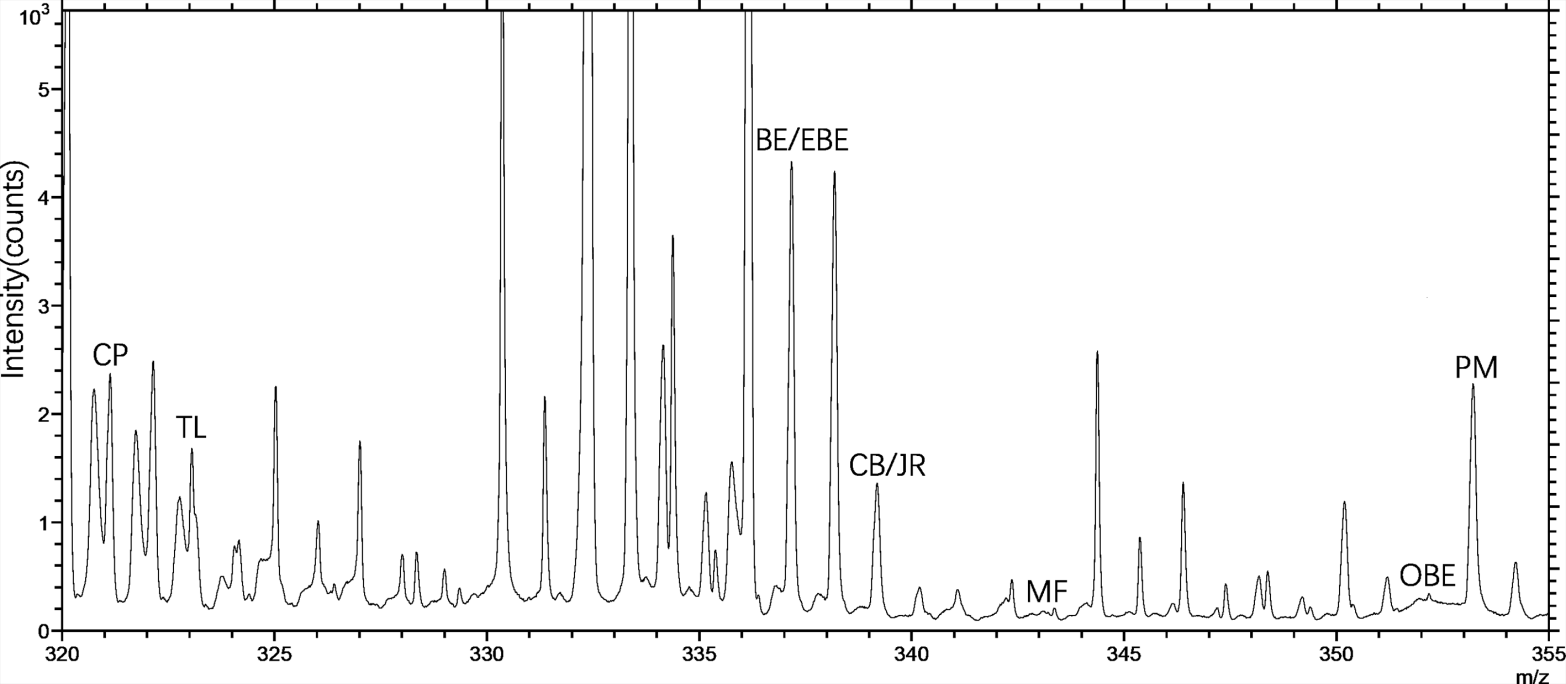
The positive TOF-SIMS spectrum of alkaloids on the cross-section of fresh *Coptis chinensis* rhizome. CP (coptisine), TL (tetrahydricheilanthifolinium), BE (berberine), EBE (epiberberine), CB (columbamine), JR (jatrorrhizine), MF (magnoflorine), OBE (oxyberberine) and PM (palmatine).

**Figure 6:**
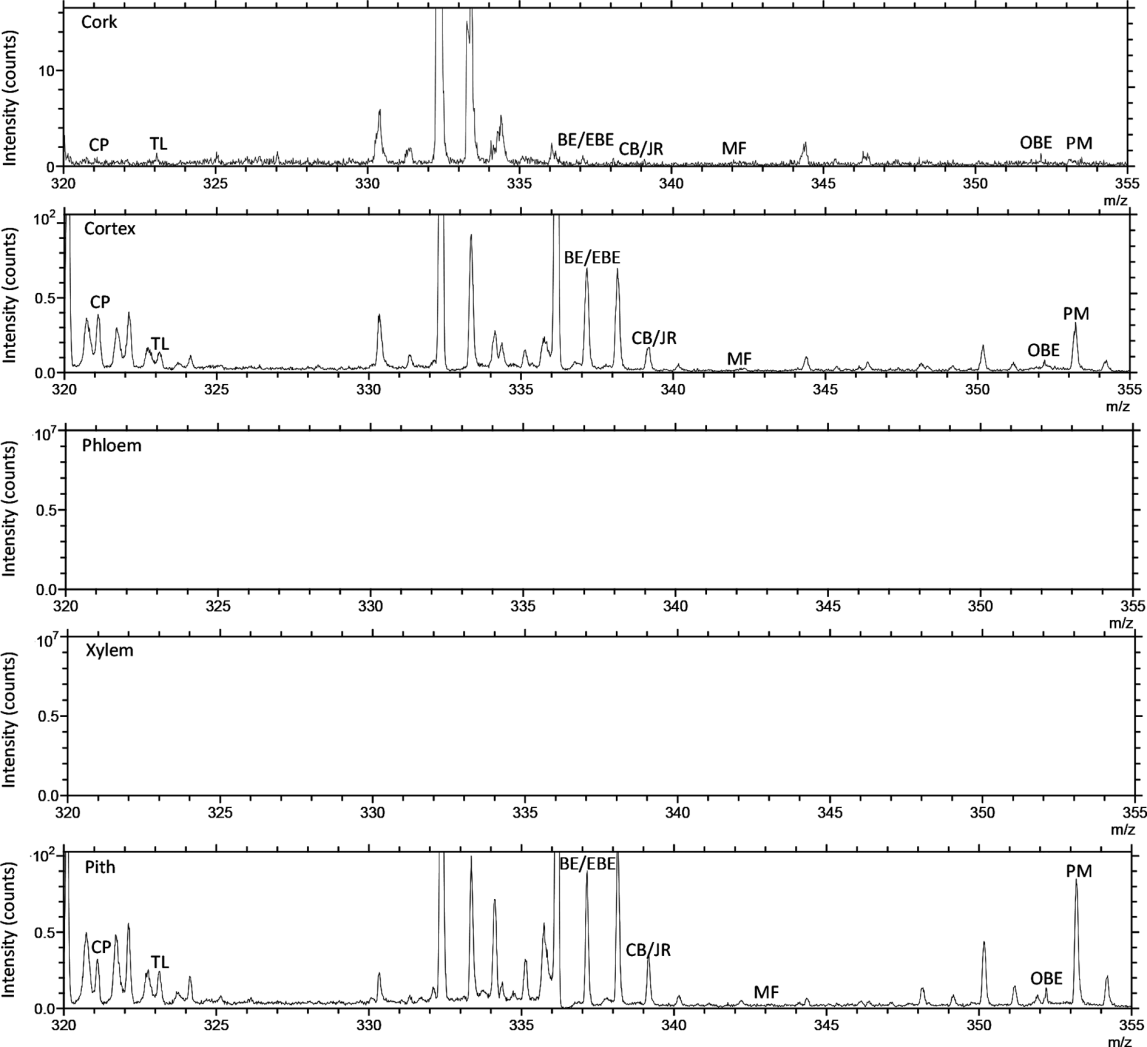
The positive TOF-SIMS ROI spectrum of alkaloids on the cross-section of fresh *Coptis chinensis* rhizome. CP (coptisine), TL (tetrahydricheilanthifolinium), BE (berberine), EBE (epiberberine), CB (columbamine), JR (jatrorrhizine), MF (magnoflorine), OBE (oxyberberine) and PM (palmatine).

TOF-SIMS imaging was used to analyze the distribution of alkaloids in the cross-section of the rhizome of the fresh CR. Before doing any chemical imaging, the mass spectrum of each molecule needed to be understood. The names and *m/z* values of the ions corresponding to the specified compounds are listed in **Table 2**. The positive ion spectrum of alkaloids on the cross-section of fresh CR is shown in **Figure 5**. The figure shows that the chromatographic peak of BE/EBE (isomers) was the highest, indicating that the content of BE/EBE was the highest. In descending order of peak height, PM, CP, TL, CB/JR (isomers), OBE, and MF. In addition, the region of interest (ROI) analysis (**Figure 6**) showed that the alkaloids of CR were mainly concentrated in the cortex and pith, followed by the cork, and almost no alkaloids were detected in phloem and xylem.

**Table 2:** The names and *m/z* values of the ions corresponding to the specified compounds.

Compound	Formula	Mass(*m/z*)	Species
MF	C_20_H_25_NO_4_^+^	343.18	[M+H]^+^
TL	C_19_H_17_NO_4_^+^	323.12	[M+H]^+^
CP	C_19_H_15_NO_4_^+^	321.09	[M+H]^+^
BE (EBE)	C_20_H_19_NO_4_^+^	337.14	[M+H]^+^
CB (JR)	C_20_H_21_NO_4_^+^	339.18	[M+H]^+^
PM	C_21_H_23_NO_4_^+^	353.20	[M+H]^+^
OBE	C_20_H_18_NO_5_^+^	352.15	[M+H]^+^

**Figure 7:**
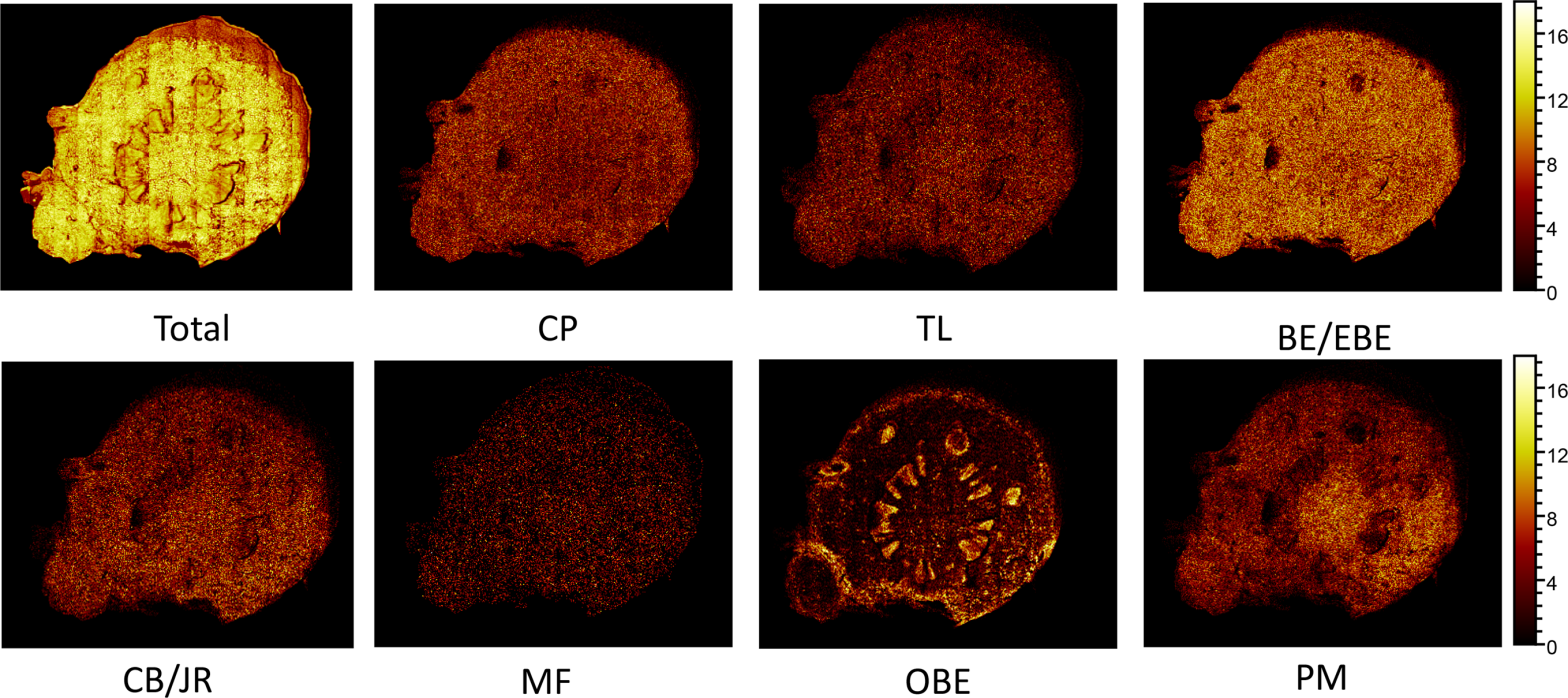
The positive TOF-SIMS images of alkaloids on the cross-section of fresh *Coptis chinensis* rhizome. CP (coptisine), TL (tetrahydricheilanthifolinium), BE (berberine), EBE (epiberberine), CB (columbamine), JR (jatrorrhizine), MF (magnoflorine), OBE (oxyberberine) and PM (palmatine).

The spatial distribution of BE/EBE, PM, CP, TL, CB/JR, OBE, and MF on the cross-section of the rhizome of CR (**Figure 7**) was drawn based on TOF-SIMS imaging technology. The intensity was represented by a color scale, where bright colors correspond to high intensity, and dark colors correspond to low intensity. For each image, the color scale was normalized to the brightest pixel. The numbers below the image refer to the ion, the intensity of the brightest pixel (MC), and the corresponding image’s total count (TC).

TOF-SIMS imaging revealed that BE/EBE, CP, PM, CB/JR, TL, and MF were scattered throughout the cross-section. Observing the intensity of the spots on the mass spectrum, it could find that the content of BE/EBE was the highest, followed by BE, CP, PM, CB/JR, and MF could only be seen as faint spots scattered. PM distributed more in the pith than in other parts. OBE was mainly concentrated in the cork and xylem rays of CR.

The TOF-SIMS was also employed to detect fresh CR samples with 4-year, 5-year, and 6-year growth from the same origin. The experimental results are shown in **Figure S1** in **Supplementary Material** and **Table 3**. **Table 3** shows the number of ions of the target peak at different growth ages. **Figure S1** shows the number of ions in **Table 3** as a visual diagram. From the intuitive results, the distribution of the alkaloids in the 5-year CR and 6-year CR was consistent with the 4-year growth CR. Comparing the TC values of each alkaloid in the different growth years CR, it was found that 4-year growth CR > 6-year growth CR > 5-year growth CR. After normalizing the TC and MC of the target compound, the relative intensities of the molecule’s peak of each compound were 6-year growth CR > 5-year growth CR > 4-year growth CR.

**Table 3:** The number of ions of the target peak at different growth ages of *Coptis chinensis* rhizome.

Compound	Formula	4 years			5 years			6 years		
		Area(counts)	Deviation(ppm)	MC/TC(100%)	Area(counts)	Deviation(ppm)	MC/TC(100%)	Area(counts)	Deviation(ppm)	MC/TC(100%)
CP	C_19_H_15_NO_4_^+^	277636	-17.8	0.194	174745	-40.5	0.237	231146	-32	0.301
TL	C_19_H_17_NO_4_^+^	137017	-13.9	0.0957	65195	-41.1	0.0883	80440	-47.4	0.105
BE (EBE)	C_20_H_19_NO_4_^+^	511067	31.3	0.357	283607	-5.9	0.384	350099	28.3	0.455
CB (JR)	C_20_H_21_NO_4_^+^	159132	36.1	0.111	75082	-4.8	0.102	83429	23.2	0.108
MF	C_20_H_25_NO_4_^+^	19629	-22.6	0.0137	4293	-0.4	0.00581	14760	11	0.019
OBE	C_20_H_18_NO_5_^+^	181751	-36.3	0.127	218284	48.6	0.296	244301	48.3	0.318
PM	C_21_H_23_NO_4_^+^	244433	44.8	0.171	87614	-31	0.119	101009	48.8	0.131
Total		143180810			73835009			76900157		

Deviation (accuracy of target peak m/z); MC (the maximum count); TC (the total count).

### 3.3 Quantification of the major constituents of CR by UPLC-QQQ-MS

**Figure 8:**
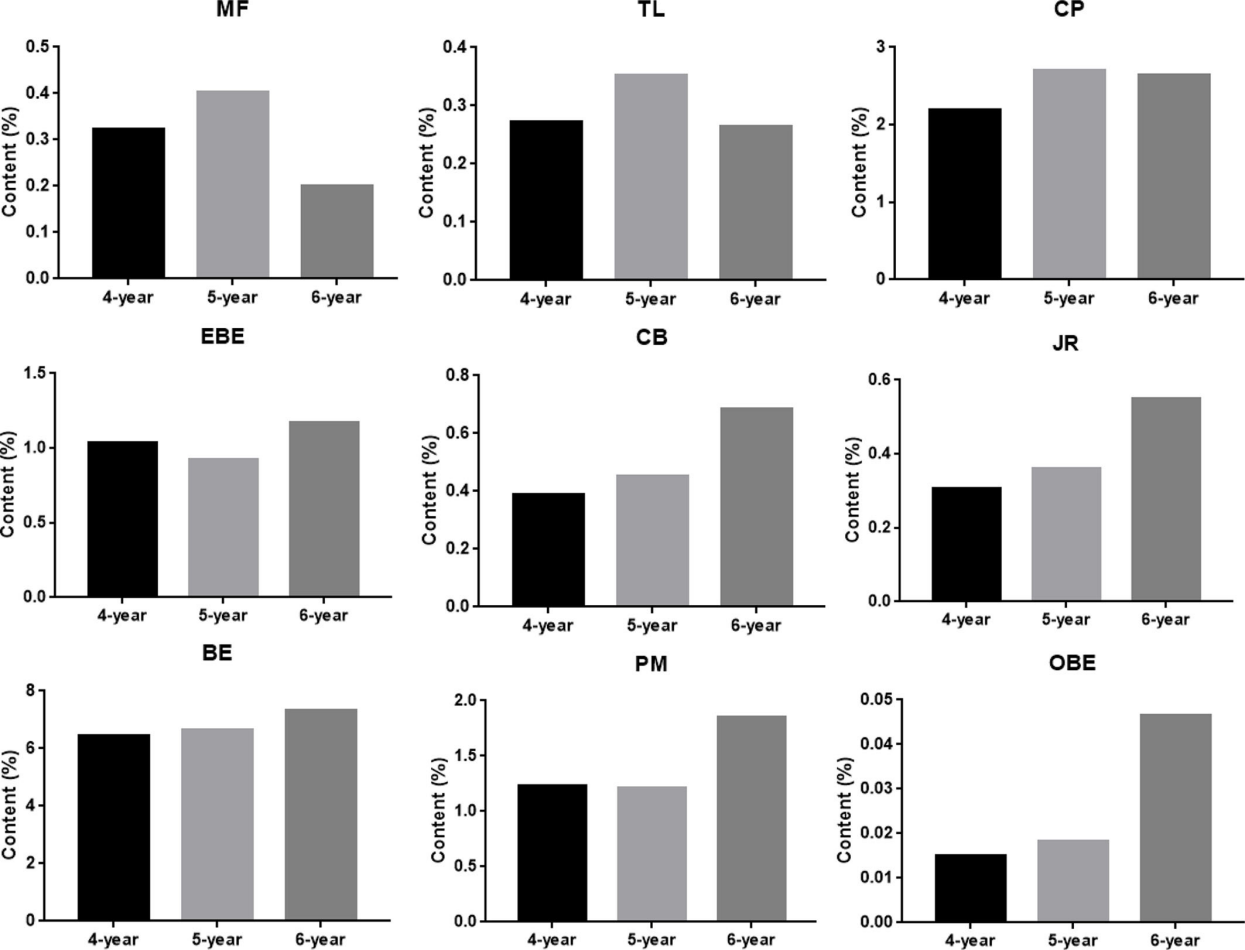
The content of nine alkaloids in *Coptis chinensis* rhizome measured with UPLC-QQQ-MS/MS. MF (magnoflorine), TL (tetrahydricheilanthifolinium), CP (coptisine), EBE (epiberberine), CB (columbamine), JR (jatrorrhizine), BE (berberine), PM (palmatine) and OBE (oxyberberine).

The results of quantitative research were used to demonstrate the credibility of TOF-SIMS results. **Figure S2** in **Supplementary Material** shows the MRM chromatogram of 9 alkaloids of CR. The calibration curves of 9 alkaloids showed good linearity between peak area and concentration (r> 0.999) (**Table S1** in **Supplementary Material**). In addition, the values of LOD and LOQ are listed in **Table S1**. As shown in **Table S2** in **Supplementary Material**, the precision [Relative Standard Deviation (RSD)] was in the range of 1.36 to 4.72%; the RSDs for repeatability and stability were <4.87% and 4.57%, respectively. The recoveries of 9 compounds ranged from 97.5% to 101.5% (RSD ≤ 4.07, **Table S2**). Subsequently, the optimized method was applied to analyze 9 compounds in 9 batches of CR. The content results are shown in **Figure 8**. The samples used in this study are 4-year, 5-year, and 6-year CR, collected from 3 places in Sichuan Province, and the harvest time was September and November. The experimental results showed that the contents of most compounds in CR increased along with the increase in growth years, while the contents of MF and TL were the highest in 5- year CR.

## 4 Discussion

The distribution of pharmacologically active ingredients in plant varieties affects the quality of TCM and the curative effect of medicinal materials. The types and contents of alkaloids of CR in different growth environments and growth stages vary greatly. Previous studies only focused on chemical compounds (Li et al., 2012; Meng et al., 2018; Hao et al., 2020), pharmacological activities (Hong et al., 2012; Kim et al., 2016; Akiyama et al., 2019; Ma et al., 2019; Teklemichael et al., 2020), molecular biology (Kitamura et al., 2018; Le et al., 2020; Chen et al., 2021), and product specifications [30] of CR, with few reporting the internal relationship between appearance characteristics and quality of medicinal materials.

Yi et al. (Yi et al., 2015) used laser microdissection and liquid chromatography-mass spectrometry (LMD-LC-MS) to detect six alkaloids in dissected sections of CR. The results showed that the content of BE was the highest, and the content of MF was the lowest in CR. Alkaloids were detected in the cork, cortex, and pith. This result was almost the same as our test result. But, LMD-LC-MS could not directly observe the distribution and accumulation of compounds on the surface of plants.

In previous studies, TOF-SIMS has proven to be an excellent tool for *in-situ* research of substances in plants (Pacholski and Winograd, 1999; Tyler et al., 2016). This study investigated the possibility of using TOF-SIMS as a new method for detecting and locating active compounds in TCM. UPLC-QQQ-MS/MS verified the results of TOF-SIMS. In the TOF-SIMS experiment, all the compounds to be tested could be identified and mapped with minimal sample preparation and labeling. The ability of TOF-SIMS to simultaneously image biological molecules of interest provided a powerful tool for studying plant physiology. However, the semi-quantification of the ionic intensities of TOF-SIMS spectra was still controversial, mainly due to different factors that affect the ionic intensities, such as the chemical composition of the sample matrix, morphology, matrix-primary ion interaction, instrument transmission, and detector response (Magnusson et al., 2008). In this study, the content of different compounds in CR is related to the ion signal intensity of the TOF-SIMS chromatogram in the same sample section. For example, **Figure 5** visually shows the ion signal strength of the seven compounds (BE/EBE > CP > PM > OBE > CB/JR >TL>MF) in CR, which was consistent with the total count of SIMS images (**Figure 7**). The UPLC-QQQ-MS/MS method was used to verify the content results of nine compounds in CR sample in **Figure S2**. Interestingly, the content of nine compounds in the sample by the UPLC-QQQ-MS/MS method was also consistent with the results of TOF-SIMS detection. Therefore, similar results were obtained for the same sample section even if the TC did not normalize the individual peaks (Ostrowski et al., 2007). Then, was the content between different CR samples also related to the ion signal strength of TOF-SIMS? The answer is no. Taking berberine as an example, the content measured by UPLC-QQQ-MS/MS method showed that the content of berberine increased along with the increase of the growth year of the sample (6 > 5 > 4). This conclusion was consistent with our empirical knowledge. However, from the TOF-SIMS spectrum, the signal intensity of BE was not consistent with sample content and growth year. According to the TOF-SIMS spectrum (**Figure S1**), the TC value of BE in the 5-year CR sample was lower than that in the other years. The inconsistency between UPLC-QQQ-MS/MS assay and TOF-SIMS imaging was speculated as the increased CR lignification resulting in weaker ionization of compounds by Bi^3+^ ions in thick-walled cells than that in parenchymal cells. The molecular ion peaks of different compounds were analyzed by the normalization method to avoid the error of the visual analysis due to the degree of ionization. The normalization can reduce the intensity difference between individual spectra caused by topography (Ostrowski et al., 2007). The TC results of the target compounds of CR in different growth years are shown in **Table 3**. After normalizing the TC of the target compound with the total TC, the relative intensity of each compound was compared. As the growth year of CR increased, each compound’s relative intensity increased, similar to the UPLC-QQQ-MS/MS detection result. For the samples of CR, the ionization effects of different plant tissue structures on the compounds are relatively the same in the same sample. Therefore, TOF-SIMS can display the distribution of compounds in different tissue structures and reflect ionization in different tissue structures. On the contrary, TOF-SIMS can only show the distribution of compounds in different tissue structures of different samples but cannot reflect the content. Many factors affect the ionization of material compounds.

## 5 Conclusion

There are many kinds of medicinal plants, and their sources and distribution are wide. Traditional identification methods have disadvantages such as inaccuracy, complicated operation, and time-consuming. Therefore, there is an urgent need for an accurate, fast, and simple method for species identification and evaluation of the quality of TCMs. For the first time, a new and stable TOF-SIMS method has been developed for the rapid and solvent-saving determination of TCMs. This study performed TOF-SIMS and UPLC-QQQ-MS/MS to detect CR at different growth years. TOF-SIMS effectively tested different chemical markers of different growth years and different parts of CR. TOF-SIMS can also be used to develop species markers of medicinal plants, pesticide residues and heavy metal residues in Chinese medicinal materials, and even the other quality evaluation systems of new natural products and their related biosynthetic pathways.

## Data availability statement

The original contributions presented in the study are included in the article/**Supplementary Material**. Further inquiries can be directed to the corresponding authors.

## Author contributions

FH: Methodology, Formal analysis, Data Curation, Writing - Original Draft, Writing - Review & Editing, Visualization. Y-FH: Data Curation, Investigation, Formal analysis, Resources. WD: Investigation, Resources. X-YQ: Investigation, Resources. J-GL: Data analysis. LC-C: Data analysis. W-HL: Validation, Formal analysis. D-MS: Validation, Data Curation. MW: Validation, Supervision. S-YX: Investigation, Resources. YX: Data curation, Methodology. LL: Conceptualization, Supervision, Writing - Review & Editing, Project administration. HZ: Conceptualization, Methodology, Supervision, Writing - Review & Editing, Project administration. All authors contributed to the article and approved the submitted version.
