# Supplementary material for: The localization of the alkaloids in Coptis chinensis rhizome by time-of-flight secondary ion mass spectrometry
Source: Front Plant Sci. 2022 Dec 23;13:1092643. doi: 10.3389/fpls.2022.1092643 (PMC9816869; doi:10.3389/fpls.2022.1092643)
Supplement: Supplementary file 1 [file DataSheet_1.docx]

Supplementary Material

# Supplementary Figures and Tables

## Supplementary Figures


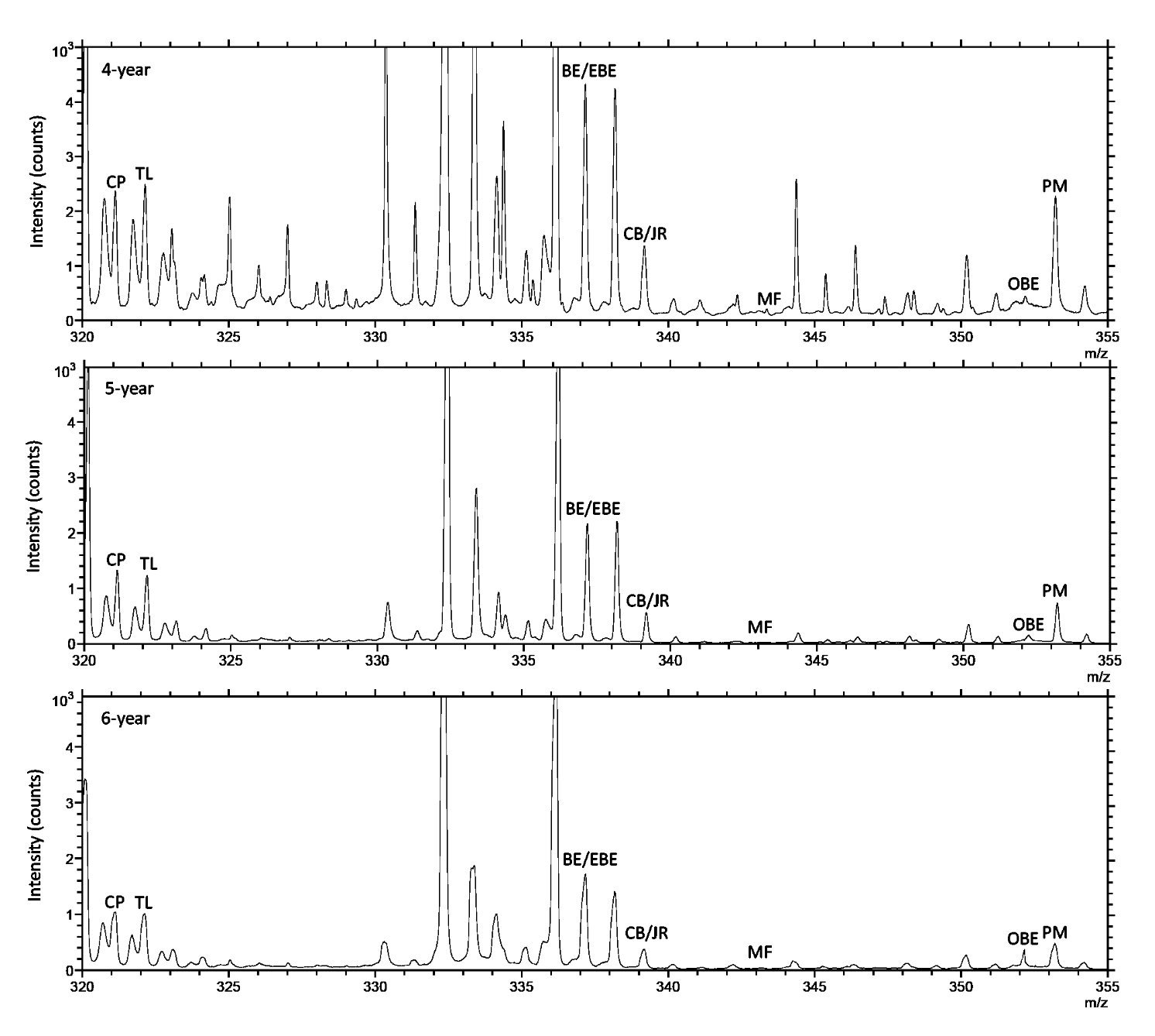


**Figure S1.** The positive TOF-SIMS spectrum of alkaloids on the cross-section of fresh *Coptis chinensis* rhizome with different growth years. CP (coptisine), TL (tetrahydricheilanthifolinium), BE (berberine), EBE (epiberberine), CB (columbamine), JR (jatrorrhizine), MF (magnoflorine), OBE (oxyberberine) and PM (palmatine)





**Figure S2.** The MRM chromatogram of 9 alkaloids of *Coptis chinensis* rhizome by UPLC-QQQ-MS/MS. A standard mixture and B CR sample. MF (magnoflorine), TL (tetrahydricheilanthifolinium), CP (coptisine), EBE (epiberberine), CB (columbamine), JR (jatrorrhizine), BE (berberine), PM (palmatine) and OBE (oxyberberine).

## Supplementary Tables

**Table S1.** Regression equation, linear ranges, LOQ and LOD of the nine compounds in *Coptis chinensis* rhizome

| Compound | Calibration Curve | r | Liner Range  (μg/mL) | LOQ  (ng/mL) | LOD  (ng/mL) |
| --- | --- | --- | --- | --- | --- |
| MF | Y=1162.9X+642.5 | 0.9991 | 0.396-6.34 | 32.7 | 9.91 |
| TL | Y=16097X+4054 | 0.9994 | 0.336-5.38 | 2.09 | 0.697 |
| CP | Y=1503.3X+2848 | 0.9992 | 2.28-36.5 | 11.8 | 3.56 |
| EBE | Y=3009.9X+6484 | 0.9991 | 1.08-17.4 | 3.56 | 1.08 |
| CB | Y=4392.0X+2446 | 0.9990 | 0.364-5.82 | 2.27 | 0.757 |
| JR | Y=5494.5X+4736 | 0.9992 | 0.772-12.4 | 1.27 | 0.386 |
| BE | Y=2263.6X+5481 | 0.9993 | 2.02-32.3 | 3.34 | 1.01 |
| PM | Y=3195.6X+5399 | 0.9991 | 1.71-27.3 | 3.52 | 1.07 |
| OBE | Y=28852xX+3347 | 0.9930 | 0.0893-1.43 | 1.16 | 0.372 |

**Table S2.** Precision, repeatability, stability, and recovery of the nine compounds in *Coptis chinensis* rhizome

| Compound | Precision | Repeatability | | Stability | Recovery | |
| --- | --- | --- | --- | --- | --- | --- |
|  | RSD%(n=6) | Conc.(mg/g) | RSD%(n=6) | RSD%(n=6) | Mean(%) | RSD%(n=6) |
| MF | 4.64 | 5.93 | 2.81 | 4.03 | 100.9 | 3.03 |
| TL | 4.72 | 22.8 | 2.53 | 4.11 | 100.9 | 1.71 |
| CP | 2.24 | 22.1 | 1.91 | 4.45 | 101.5 | 3.86 |
| EBE | 4.58 | 7.37 | 4.55 | 4.57 | 101.1 | 1.07 |
| CB | 2.26 | 6.02 | 2.64 | 3.88 | 97.5 | 1.36 |
| JR | 1.66 | 5.26 | 3.12 | 4.71 | 100.6 | 3.29 |
| BE | 1.36 | 66.5 | 0.724 | 4.32 | 100.5 | 1.15 |
| PM | 1.53 | 18.0 | 2.92 | 4.53 | 98.9 | 2.53 |
| OBE | 2.49 | 0.925 | 4.87 | 4.31 | 98.9 | 4.07 |
